# HTLV-1 HBZ protein inhibits IRF3-mediated innate immune responses

**DOI:** 10.1186/1742-4690-8-S1-A99

**Published:** 2011-06-06

**Authors:** Renée N Douville, Stéphanie Olière, Patrick L Green, Rongtuan Lin, John Hiscott

**Affiliations:** 1 Dept. of Medicine, McGill University / Lady Davis Institute, Montreal, Quebec, Canada H3T 1E2; 2 Dept. of Veterinary Biosciences, Ohio State University, Columbus, Ohio, 43210, USA; 3 Vaccine and Gene Therapy Institute – Florida, Port St. Lucie, Florida, 34987, USA

## 

The bZIP factor (HBZ) is an HTLV-1 regulatory protein encoded by anti-sense transcription of the HTLV-1 genome. HBZ mRNA expression correlates with clinical disability in HAM/TSP patients – and can be reversed by interferon (IFN) therapy. Sporadic evidence suggests that HBZ may have a negative role on interferon signalling. Activation of IRF3-dependent IFN signalling – either direct induction of IFNβ, viral restriction factors or interferon stimulated genes (ISGs) – is crucial for TLR and RLR mediated antiviral response. Thus, we sought to determine whether HBZ can impair IRF3-mediated innate immune responses. Over-expression of active forms of RIG-I, MAVS, TBK1, IKKε or IRF3 alone drive an antiviral response – however, in the presence of an HBZ expression vector, IFNβ responses were abrogated by 50-70%. In contrast, HBZ enhanced IRF7-dependent responses. In confirmation, both PBMC and human astrocytes transfected with HBZ and subsequently stimulated with IFN-triggering ligands (LPS, PolyI:C, VSV, Sendai virus and HTLV-1 virions), exhibited impaired IRF3-dependent signalling as compared with controls. As IRF3 is known to bind other bZIP proteins, further studies are underway to delineate the nature of IRF-HBZ interactions. Identifying such a mechanism may explain an enhanced risk of neurologic infection, as we show that chronically HTLV-1 infected astrocytes gradually increase and maintain long-term HBZ expression. Defining the immunomodulatory properties of HTLV-1 HBZ protein will provide a vital contribution toward understanding clinical outcome and risk of opportunistic infection associated with HTLV-1 infection.

